# Effect of Different Bond Parameters on the Mechanical Properties of FRP and Concrete Interface

**DOI:** 10.3390/polym12112466

**Published:** 2020-10-24

**Authors:** Comfort Mensah, Zhenqing Wang, Alex Osei Bonsu, Wenyan Liang

**Affiliations:** College of Aerospace and Civil Engineering, Harbin Engineering University, Harbin 150001, China; comfortmensah@hrbeu.edu.cn (C.M.); alexoseibonsu@hrbeu.edu.cn (A.O.B.); liangwenyan@hrbeu.edu.cn (W.L.)

**Keywords:** FRP laminate, concrete, bonding interface, bond–slip behavior, mechanical property, externally bonded reinforcement

## Abstract

This paper presents double shear tests performed to investigate factors influencing the bond behavior between basalt fiber-reinforced polymer (BFRP), glass fiber-reinforced polymer (GFRP) laminate, and concrete blocks. In detail, thirty-six twin concrete blocks strengthened with the aforementioned FRP types were tested to evaluate the influence of FRP length, width, and thickness, and their bonding behavior. The 2D-DIC (digital image correlation) technique and several strain gauges bonded along the laminate were used to measure the strain distributions of the FRP-to-concrete interface. The failure mode, ultimate load, load–slip, strain distribution, and bond–slip relationships between the laminates and concrete were analyzed. Furthermore, bond–slip curves were compared with some other existing literature models. The results from the experiment showed that the ultimate load, peak bond stress, and slip increased with the increase in the BFRP and GFRP laminates length, width, and thickness. The values of peak shear stress and the corresponding maximum shear slip were significantly different because of the above-mentioned factors’ influence on them. The bond interface that contributes to the bearing of the shear load may grow to an extent and later shift from the loaded end when debonding progresses. Finally, the fractured surfaces of the failed FRP laminates were examined using scanning electron microscope (SEM), revealing that FRP rupture, debonding in concrete, and debonding in an adhesive–concrete interface were the main failure types.

## 1. Introduction

The wide use of externally bonded fiber-reinforced polymer (FRP) laminates (FRP sheets or FRP plates) for the strengthening, retrofitting, and repairing of ailing reinforced concrete structures has gradually become a popular area of current research in the recent decade [1,2]. This is mostly attributed to the many excellent characteristics such as high resistance to corrosion, excellent tensile strength, light weight, and high fatigue strength [3,4]. The interfacial bond behavior of FRP–concrete is vital in preventing debonding that occurs in different types of FRP-strengthened reinforced concrete structures [5,6,7,8]. Premature debonding limits the reinforcement efficacy of FRP strengthened concrete structures, as it only takes place at the lower strain rather than the ultimate strain [9]. Research studies have documented that the commonly encountered failure mode of externally bonded (EB) FRP composites strengthened concrete members is premature FRP debonding from concrete substrate caused by high-stress concentrations [10]. Therefore, it is important to develop a safe and cost-effective design of an externally bonded structure for a better understanding of the behavior between FRP-to-concrete interfaces.

The external wrapping or bonding of FRP laminates has proven to be an effective and popular method for the rehabilitation, strengthening, and retrofitting of reinforced concrete (RC) structures in both shear and flexure [11]. The effectiveness of this strengthening technique and mechanical performance of reinforced concrete structures is largely determined mostly by the bond behavior of FRP and concrete substrate. Therefore, it is necessary to analyze the quantitative bond characteristics of FRP–concrete. Several researchers have reported on the behavior of the bond interface between FRP and concrete using several techniques. Among these techniques are the double shear test [12,13,14,15], single shear test [16,17], and beam test [18,19,20,21]. The use of the single and double shear testing approach has so far become the most common owing to their flexibility in design as well as stable devices. Parameters that affect the bond behavior of FRP-to-concrete joints have been evaluated. Chajes et al. [17] have examined the effect of surface preparation, different kinds of adhesives, as well as concrete strength utilizing the single shear testing procedure. de Azevedo et al. [22] conducted experimental studies on the technological performance of açaí natural fiber-reinforced cement-based mortars. Azevedo et al. [23] also conducted research on the development of mortar for laying and coating with pineapple fiber. Marvila et al. [24] investigated the durability of coating mortars containing açaí fibers. Amaral et al. [25] proposed a new methodology that aims at the partial replacement of sand, which is used as aggregate in mortars production, by ornamental stone waste, which is generated from cutting and others productive processes. Maeda et al. [15] conducted a thorough experimental analysis on the bond behavior of FRP and concrete joints. The influence of various parameters such as the bond length, number of plies, and FRP sheet type on the bond behavior have been discussed and analyzed. The experimental studies revealed that the performance of the bond joints strongly depends on the strength of the concrete and the FRP-to-concrete member width ratio. The cracking of concrete under shear has been one of the key failure mechanisms of FRP–concrete joints during shear testing, which usually occurs at several millimeters from the adhesive–concrete interface. Moreover, several models were developed to evaluate the behavior of the bond between FRP-to-concrete joints based on results obtained from experiments [5,26], numerical simulation results [27], and the theory of mechanics of fracture.

The tensile force in the laminate declines gradually toward the fixed end of the laminate in the tensile experiment. When the loads are higher, the tensile force tends to increase within the initial bond region. Once the bond strength has been achieved, debonding starts to propagate, bond strength extents further from initial bond region, and thus, the effective bond region moves to different locations. As a result, only a portion of the bond becomes active, and the bond strength may not always increase with increasing length. In this case, it is impossible to reach the ultimate tensile strength of the laminate. The explanation above is the major distinction between the externally bonded reinforcement and internal reinforcement in which the ultimate tensile strength of the reinforcement could be reached with a considerably longer anchoring length. Moreover, the majority of current research studies concentrate on predicting the ultimate load as well as the effective bond length [1]. Meanwhile, Oliveira et al. [28] conducted a study on the use of waste collected from wind turbine blade production as an eco-friendly ingredient in mortars for civil construction. de Mendonça Neuba et al. [29] also studied the promising mechanical, thermal, and ballistic properties of novel epoxy composites reinforced with cyperus malaccensis sedge fiber.

Currently, a significant number of studies reported that the effects of the bond between concrete and FRP laminates on the failure of adhesively bonded joints are predicted mostly by analyzing a number of factors that can affect their relationship. These factors include the concrete compressive and tensile strengths, FRP bond length and width, adhesive properties, FRP axial stiffness and thickness composition, FRP and concrete width ratio, type of FRP sheets, and surface preparation. Therefore, as a consequence, the mechanical behavior of the interface between the concrete and FRP laminates has to be understood and revealed. Zheng et al. [30] experimentally studied the static and fatigue bond behaviors of the bonded interface between carbon fiber laminate (CFL) and concrete using the double-shear test. The influence of various parameters such as the bond length, bond width, type of load (monotonic or fatigue loading), and different fatigue load levels was discussed. Yang and Li [31] investigated the bonding mechanical properties of sprayed FRP and concrete substrate. The double shear tests were performed with three parameters: fiber volume fraction, FRP thickness, and bond length on the bond behavior were analyzed. Even though a considerable amount of studies has been conducted to quantitatively analyze these factors [32,33,34,35], the quantitative features have proven difficult to comprehend, as the cumulative impact of these factors influence the bond characteristics. Additionally, although several models have been developed concerning the bond characteristics between concrete and FRP laminate [36,37,38,39], a right bond stress–slip model is yet to be acknowledged in general because of its complex nature and countless parameters.

Studying the mechanical performance of concrete structures strengthened with basalt fiber (BFRP) and glass fiber (GFRP) composites is a prominent area of current research with many important aspects that needs to be addressed. This paper investigates the factors that influence the performance of the bond between the FRP laminate and concrete interface through double shear testing. The main parameters analyzed in this paper were FRP bond length, FRP width, and FRP thickness. To achieve this, two kinds of FRP (i.e., BFRP and GFRP) were used for the preparation of FRP laminates. Then, the specimens were strengthened with the different laminates to cover a wide range of FRP axial rigidities. Ncorr, an open-source 2D-DIC Matlab program, as well as the conventional strain-measuring device (e.g., strain gauge) were employed to measure the strains distribution over the surface of the laminate. Of these, 36 double shear specimens were instrumented to examine the effects of factors on the bond interface with respect to the failure mode, the ultimate load, load–slip, the variation in axial strain distribution, and the shear stress distribution, through which the bond–slip relationship of FRP laminate–concrete interfaces was documented and studied.

## 2. Materials and Methods

### 2.1. Materials Properties

The raw materials used during casting of the concrete blocks include Portland cement with a grade of 42.5 per the Chinese standard code [40], and was provided by Yatai Cement Co., Ltd. (Harbin, China). Locally available coarse aggregates with grain size of 5–20 mm, fine aggregates of river sand, and laboratory tap water was used. They were acquired from a local vendor also in Harbin (China). The strength grade of the concrete used was C30, which is consistent with the Chinese Code for the design of concrete structures [41]. The concrete blocks were removed 24 h after casting and cured for more than 28 days at a relative humidity of 98% in the laboratory. The average compressive strength obtained from compression tests of three standards cubes of 150 mm × 150 mm × 150 mm was experimentally measured as 46.09 MPa.

Two FRP types (basalt fiber and glass fiber) were used as the reinforcement material in this study with thicknesses of 0.42, 0.41, 0.81, and 0.83 mm. BFRP and GFRP tensile specimens were fabricated in accordance with ASTM D3039 for their mechanical properties [42]. The average tensile strength and elastic modulus are 681.50 MPa, 373.48 MPa, 16.13 GPa, and 24.80 GPa respectively. The adhesive used for binding FRP laminate to the concrete surface consisted of two-component epoxy adhesive with a shear strength of 20 MPa and working temperature of −60 °C + 120 °C according to the manufacturer’s datasheet. The two-component epoxy adhesive was produced by Yasong Company (Dongguan, China).

### 2.2. Specimens Preparation

**Figure 1:**
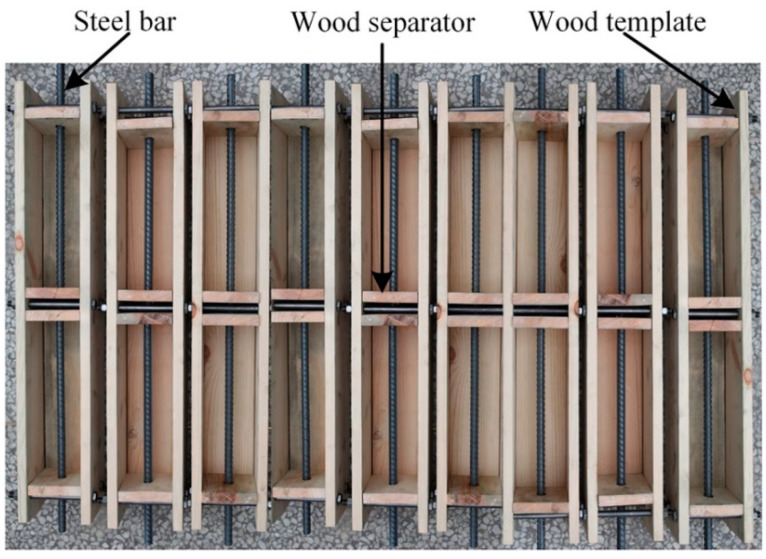
Manufactured wooden molds for casting concrete with steel rod inserted.

**Figure 2:**
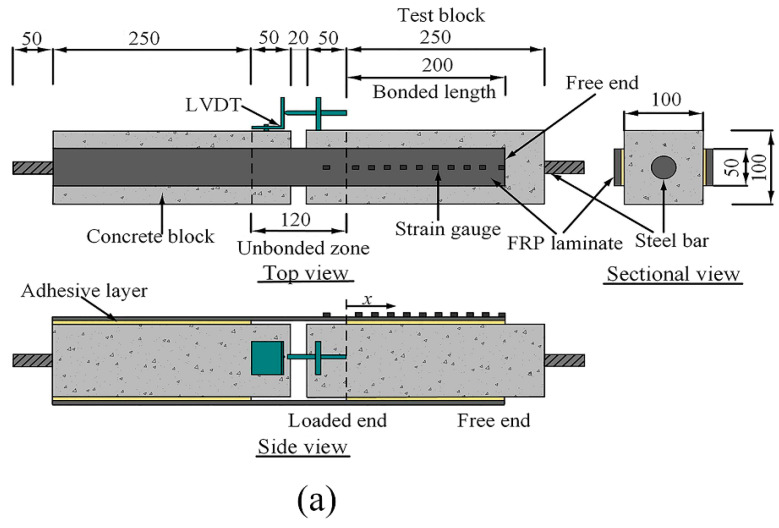

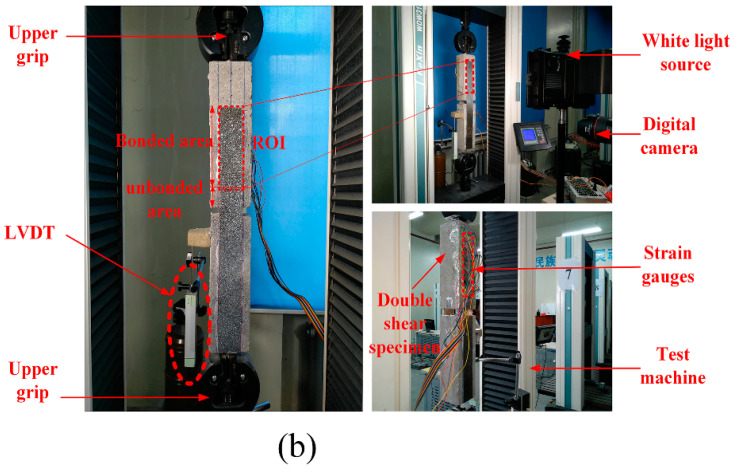
(**a**) Detailed dimensions of the double shear test specimen and (**b**) experimental setup layout (all units in mm).

Double shear specimens have been used to analyze the influence of FRP bond length, width, and thickness on the bond behavior between FRP and concrete. It should be noted that the interface usually tends to refer to the interfacial part of the FRP and concrete joint, such as the FRP sheet, adhesive, and a thin layer of concrete underneath the adhesive. The materials mentioned in Section 2.1 were utilized to fabricate the needed concrete and FRP laminate test specimens. The test specimens consisted of two equal concrete blocks (100 mm × 100 mm × 300 mm), which were prepared by first and foremost manufacturing a certain number of wooden molds. Figure 1 shows the manufactured wooden molds used in casting concrete blocks. Steel rebars with a diameter of 16 mm cut to desire lengths were inserted into the middle of the concrete blocks in which the hydraulic testing machine is able to apply tension forces at the outer ends of the individual blocks. The steel rebars were not connected at the center, meaning that the two concrete blocks are only connected through the BFRP/GFRP reinforcement. In total, 36 specimens with two types of FRP (basalt fiber and glass fiber) strengthening systems in eight sets were cast to be employed in performing the double shear tests. Each set had three identical specimens to reduce any uncertainties. Figure 2a displays the detailed information of the double shear specimens. Different thicknesses, lengths, and widths of laminates were bonded with a two-component epoxy adhesive on the opposite sides of the concrete blocks. The laminates were left unbonded across a central area of 100 mm, where the two concrete blocks were joined together.

**Figure 3:**
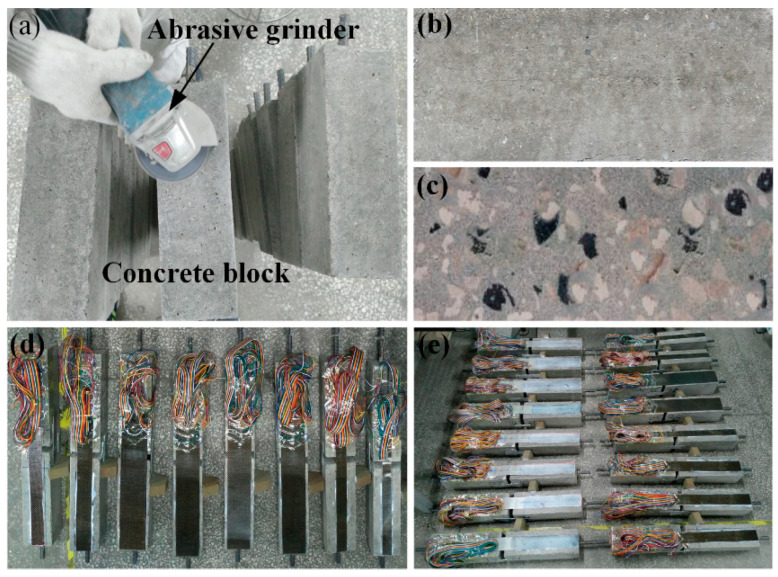
Preparation of concrete surface; (**a**) grinding of concrete surface, (**b**) unprepared surface, (**c**) prepared surface, (**d**,**e**) fabricated fiber-reinforced polymer (FRP)–concrete specimen produced.

In order to ensure maximum possible bonding, the top surfaces of the concrete blocks were grounded using a mechanical abrasive grinder. The dirt and loose particles were eliminated using a vacuum cleaner, as shown in Figure 3a. Then, the concrete surface was scrubbed with an absolute acetone wetted cloth; thus, a rough and clean surface was formed and demarcated with location lines. Then, the two-component epoxy adhesive was evenly coated on the newly treated concrete surface. Then, the FRP laminate was bonded onto each concrete block by the wet layup method and kept in place with weights to avoid air bubbles between the surface and laminate. In this paper, this bonding method was consistent for all FRP laminate-reinforced concrete specimens. Figure 3b,c shows the difference between the prepared and unprepared surfaces of concrete blocks. All specimens were stored in the laboratory environment for epoxy curing for at least 10 days, as shown in Figure 3d,e.

### 2.3. Double Shear Test Setup and Instrumentation

Figure 2b shows the setup of the double shear testing. To ensure strength development in epoxy adhesive, all specimens were cured for ten days before testing under laboratory conditions. After curing, all the specimens were subjected to a double shear test using a 100 kN hydraulic tensile testing machine. The steel bars inserted into concrete blocks were being used to secure specimens in the grips of the testing machine. Thus, tensile load is applied on the BFRP/GFRP laminate while pulling the blocks apart. The BFRP/GFRP laminate that has been glued to the opposing sides of the blocks will ensure alignment between the two blocks during double shear loading. Linear variable differential transducer (LVDT) has been used to document the slip between the laminate and concrete. Each of the specimens were evenly mounted with electrical strain gauges on the bonded face at a spacing of 20 mm to record the evolution of strains across the BFRP/GFRP bonded length. A BX120-2AA strain gauge was used in the test with a resistance of 1002 ± 0.1 Ω. During the entire testing process, the gathering of data from the strain gauges and LVDT were obtained by the use of a logger and computer system. Testing was carried out under a displacement control rate of 2 mm/min until failure.

Strains on the double shear specimen surface were also measured utilizing the digital image correlation (DIC) method and compared with that of the conventional strain-measuring device (e.g., strain gauge). Deformations and strains have been determined by measuring the displacements via successive images on a textured surface. To achieve this, the surface of the specimen was sprinkled evenly with white paint. Then, a speckle pattern was produced using a black paint mist. Digital images were captured by using a Canon DSLR camera with 25 megapixels (3872 × 2592 pixels), which was placed perpendicular to the face of the specimen. The surface of the specimen was lighted with two normal white lights to achieve a uniform light intensity on the surfaces of specimens under testing. Images of each specimen undergoing deformation were automatically taken at a regular interval using a remote control. Ncorr, an open-source 2D-DIC MATLAB software package, was employed to process the recorded digital images and to calculate the strains and deformations of specimens. Ncorr is a recognized open-source software package that has implemented many DIC algorithms; more improvements and modifications have recently been proposed as well. The application of this software package has already been tested and verified by a large number of studies [43].

## 3. Testing Results and Discussions

The key test results obtained through the double shear testing between the BFRP/GFRP laminate and concrete substrate are highlighted below. Table 1 lists the full data from the experiment, which are being used in the following discussions. The specimen is identified with an alphanumeric label (e.g., B-200-50-1-3), which was based on the FRP type, bond length, width, and number of FRP sheet layers (FRP thickness). The number that comes after the last hyphen indicates the specimen number. The results and discussions are structured based on the failure mode of the specimens, ultimate load, load–slip, strain distributions in BFRP/GFRP laminate at different loading levels, and the calculated bond stress–slip relationships for all tested specimens.

### 3.1. Failure Modes and Debonding Mechanism

**Figure 4:**
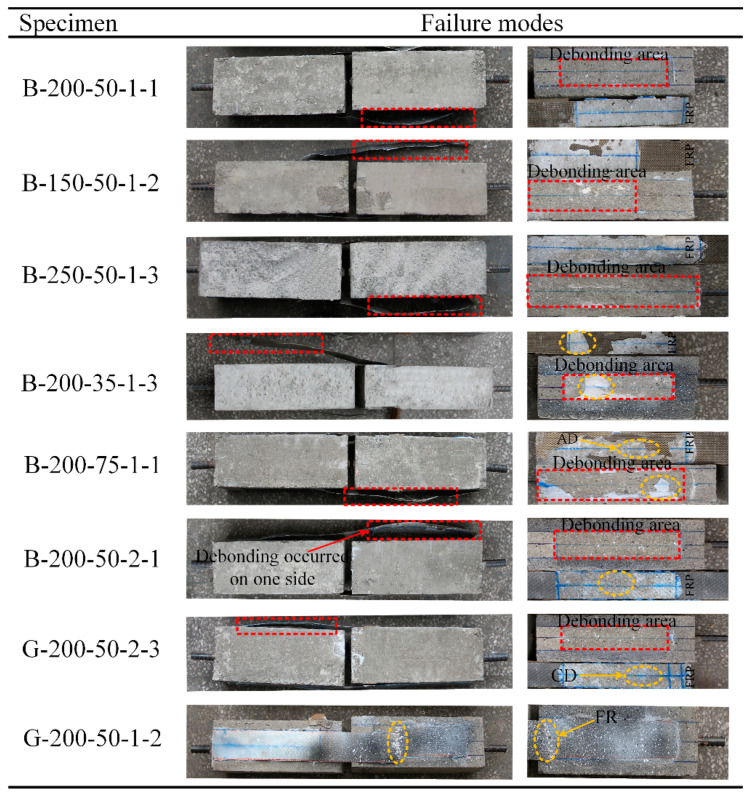
Failure modes of specimens: CD means debonding in the concrete layer, AD is debonding at the adhesive–concrete interface, and FR is FRP rupture.

Figure 4 shows the typical failure mode of the FRP–concrete bond of the specimens after testing. In this study, except for the failure of control specimens strengthened with GFRP laminate (i.e., G-200-50-1 set) which experienced FRP rupture, the failures for the remaining specimens were being caused by a variety of debonding modes. The rupture failure happened in the middle of the specimen (between the two concrete blocks) close to the loaded end, as shown in Figure 4. Specimens that failed from FRP rupture could be possibly attributed to the concentration of shear stresses in GFRP laminates that are close to the loaded end. For the specimens B-200-35-1-1, B-200-35-1-2, and B-150-50-1-2, it was observed that there was a significant amount of adhesive left intact on the surface of the concrete. This signifies the debonding failure in the adhesive–BFRP laminate interface. A thin layer of adhesive was found on the specimen B-250-50-1-3 laminate with no concrete attached, implying that the debonding failure occurred in the adhesive–concrete interface. For specimens that failed by debonding in concrete, a flake layer of concrete was attached to the FRP laminate, which is consistent with the earlier research studies [44,45]. However, the debonding failure took place on one side only, while the opposite side of the specimen bonded well. The above-mentioned failure mode resulting from different debonding patterns is due to the inhomogeneity of the concrete bonding surface. As the aggregate is much stronger than the mortar, debonding in concrete takes place in the region of the mortar, and the separation of the interface happens in the aggregate region. In addition, the prevalent debonding mode of the samples has to do with the distribution of aggregate density.

**Figure 5:**
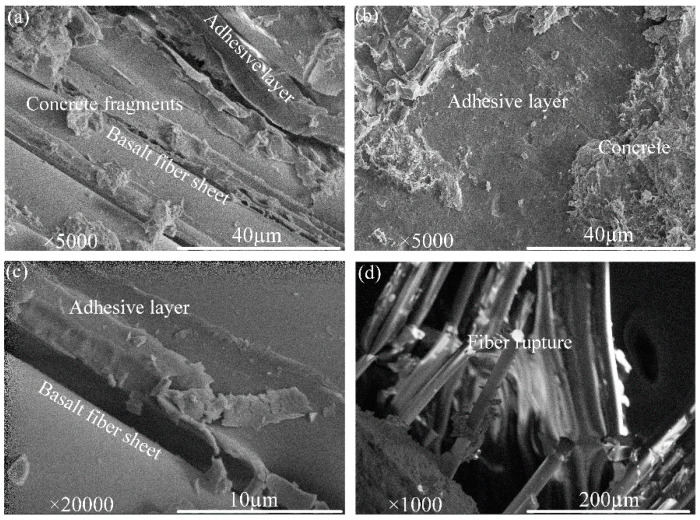
SEM observation of FRP laminates after bond test. (**a**) Matrix debris attached to basalt fiber, (**b**) concrete fragments attached to basalt fiber, (**c**) adhesion between epoxy resin and basalt fiber, (**d**) fiber rupture in G-200-50-1-3 specimen strengthened with glass fiber laminate.

To further investigate the damage mechanisms of the interface between FRP and concrete, the fracture surfaces of specimens were examined using scanning electron microscope (SEM) to detect the changes in the interface between the fiber, matrix, and aggregates. The failed samples from the bond test were extracted from the width center of the debonded laminates at different locations. Samples were examined to distinguish the changes in failure modes, and the result is shown in Figure 5. Figure 5a,c show a considerable amount of matrix debris that remained on FRP laminates in specimens B-250-50-2 and B-200-35-1. This indicates the better adhesion performance between FRP and the epoxy adhesive. The SEM image for specimen B-200-75-1 is presented in Figure 5b. It was obvious that more fragments of concrete are attached to the epoxy adhesive, indicating debonding in concrete. This observation explains the delay in debonding propagation and hence improves the load transfer between FRP laminates and the concrete substrate. Moreover, for specimens G-200-50-1 in Figure 5d, FRP rupture was dominant, which was possibly attributed to the concentration of shear stresses at the loaded end.

### 3.2. Ultimate Load and Deformation

**Figure 6:**
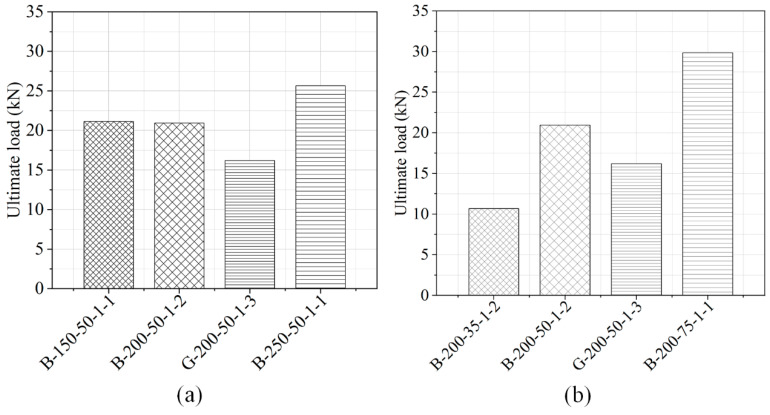

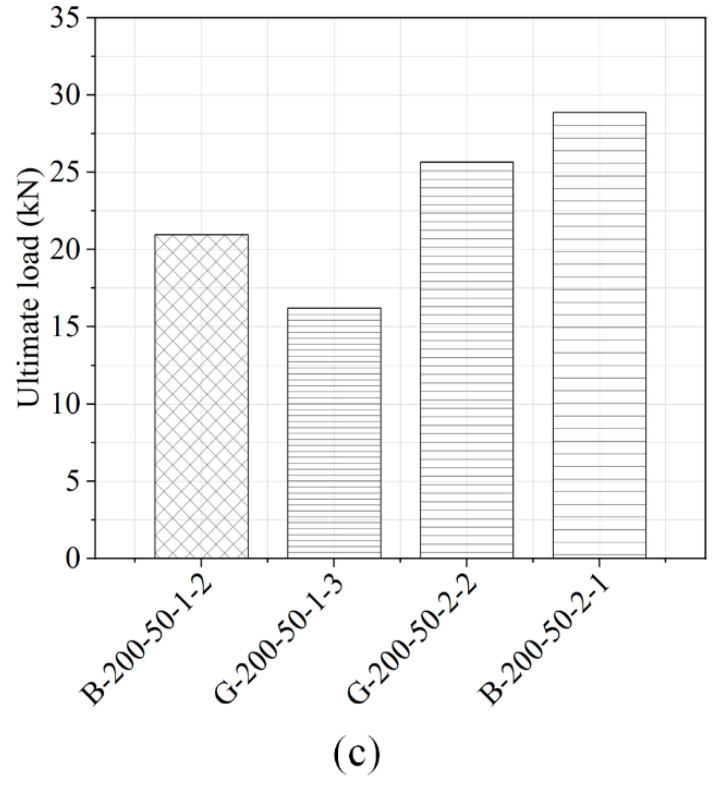
Comparison of the different factors affecting the ultimate load; (**a**) FRP bond length, (**b**) FRP width, and (**c**) FRP thickness.

The ultimate load, average ultimate load, as well as the failure mechanism are summarized in Table 1. In this experiment, the ultimate load can be described as the highest tensile load reached by the double shear specimens in the entire loading process. The failure load means the load at which the specimen failed. The control specimens strengthened with BFRP laminate had an average ultimate load of 18.03 kN. Specimen B-200-75-1-1 had the highest ultimate load, which was 29.86 kN, and specimen B-200-35-1-3 had the lowest ultimate load, which was only 7.47 kN. Hence, a wider bonding width of FRP laminate significantly improved the ultimate load of externally bonded (EB) reinforced concrete structures. Figure 6 displays the results of the three key factors such as FRP bond length, width, and thickness on the FRP–concrete interface bonding behavior.

The effects of bonding length on the ultimate load obtained from specimens with three distinct lengths (150, 200, and 250 mm) are plotted in Figure 6a. Increasing the bonding length of FRP laminate can have a major impact on the ultimate load of the specimens. A longer bonding length can improve the ultimate load. The ultimate load of B-250-50-1 specimens (250 mm bond length) was 43.0% greater than that of B-150-50-1 specimens (150 mm bond length) and also 22.4% greater than that of B-200-50-1 specimens (200 mm bond length). In addition, BFRP specimens with a 200 mm bond length had an ultimate load of 29.4%, which was much higher than the GFRP specimens with same 200 mm bond length.

The impact of FRP width on the ultimate load was evaluated by comparing the results of three different FRP widths (35, 50, and 75 mm) for a given FRP bond length, as shown in Figure 6b. It can be seen that the increase in FRP width significantly improved the ultimate load of specimens. The ultimate load for B-200-75-1 specimens (75 mm width) was 179% greater than that of B-200-35-1 specimens (35 mm width) and 42.5% greater than that of specimens with 50 mm width.

The findings on the ultimate load of specimens of various FRP thicknesses are shown in Figure 6c. As the thickness of the FRP laminate increased, the ultimate load also increased greatly as well. The ultimate load of the specimens reinforced with double FRP sheet layers (with thicknesses of 0.83 and 0.81 mm) increased by 37.8% and 58.3% higher than the specimen strengthened with only one FRP sheet layer of BFRP and GFRP, respectively. The above results correspond to similar findings of Yang and Li [31] featuring a double shear test on the bonding mechanical properties of a sprayed FRP and concrete substrate.

### 3.3. Load–Slip Relationships of FRP–Concrete Bond

**Figure 7:**
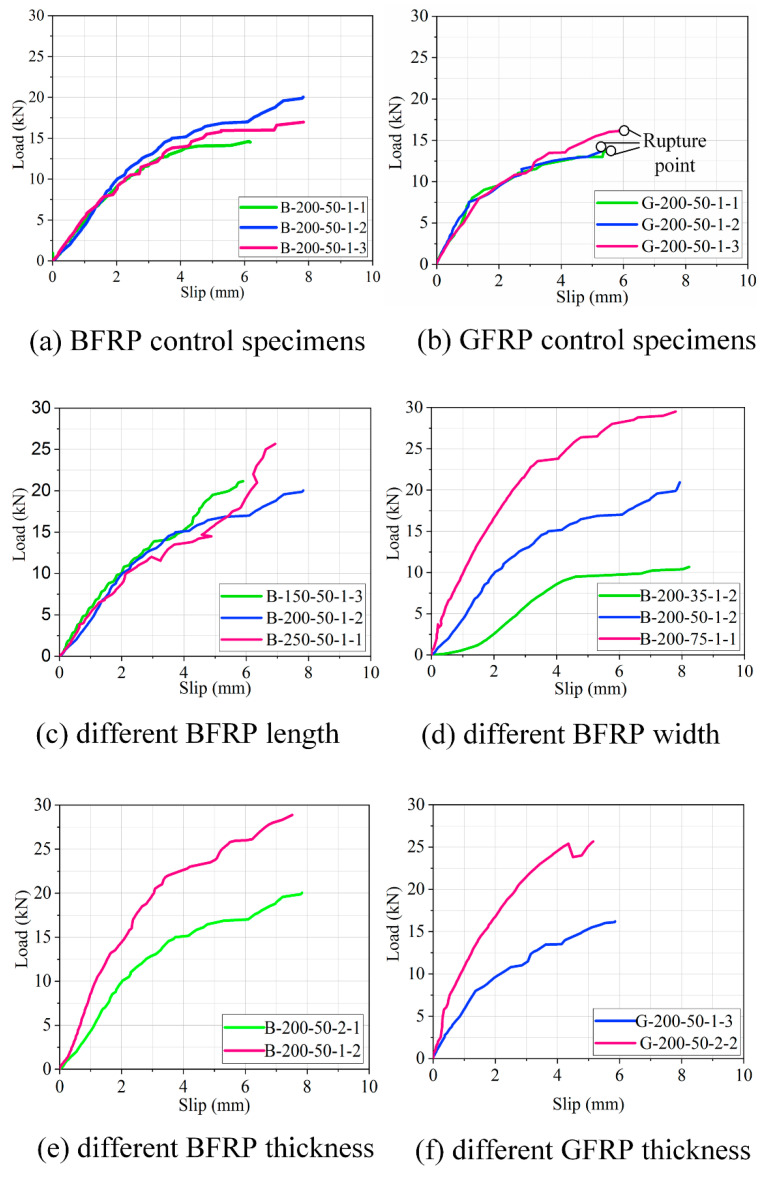
Comparison of load–slip relations of basalt fiber-reinforced polymer (BFRP) and glass fiber-reinforced polymer (GFRP) specimens.

Figure 7 illustrates load–slip relationship for the double shear specimens in which the effect of three factors are compared. In this study, the load–slip curves of the specimens G-200-50-1 set that experienced FRP rupture failure are also shown for clarification. The load–slip curves for double shear specimens in this experiment can be divided into three stages. Stage one is a linear–elastic growth stage during which the FRP laminate and concrete bonding interface remained intact. Slip increased linearly with the increase of load. In stage two, the load–slip curves increased nonlinearly, implying that the bond interface is beginning to be compromised. The relationship between the load and slip changed from a straight line to a curve. Most of the ductility is developed in stage three, introducing debonding propagation where the slip increased rapidly as the load almost levels off.

Figure 7a,b shows an almost identical load–slip curve for three repeating specimens in the B-200-50-1 set. The maximum ultimate loads of the specimen with B-200-50-1-2 and G-200-50-1-3 are larger than those of the other specimens. The three identical specimens with the same bond parameters may have apparent variations in maximum slip possibly because of the incorrect calculation of the slip.

The effect of three distinct bonding lengths of 150, 200, and 250 mm on the load–slip curves are plotted in Figure 7c. The ultimate load improved greatly by increasing the bonding length. The slip also increased as the bond length of the specimens increased from 150 to 200 mm. However, there is no obvious change in slip as soon as the bond length increased from 200 to 250 mm. The shape of the load–slip curves of B-200-50-1-1 specimen in stage one is approximately linear, indicating that the bond length has nearly no effect on stage one.

Figure 7d depicts the influence of three different FRP widths of 35, 50, and 75 mm on the load–slip curves. The pattern of the load–slip curves is nearly same, as the specimen with larger laminate experienced higher stiffness and stronger ductility. The specimens with relatively low stiffness revealed that the longitudinal slip between the FRP laminate and concrete was significantly greater, which was probably due to the much larger adhesive thickness for the FRP width group than those of the other groups.

Figure 7e,f shows the comparison of load–slip response for specimens B-200-50-1-1, B-200-50-2-1, G-200-50-1-1, and G-200-50-2-1. The thickness of the BFRP and GFRP laminate has a noticeable effect on the stiffness and load-bearing capacity of the bond performance. The ultimate load of specimens increased greatly as the thickness of the laminate increases with the slip until failure.

### 3.4. Strain Distribution in BFRP and GFRP Laminates

**Figure 8:**
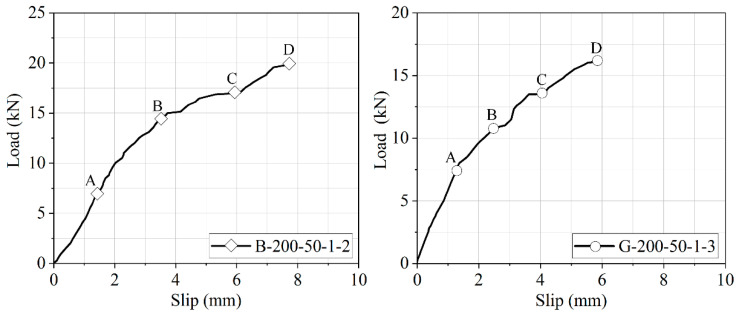
Load–slip response for specimens and its loading points of B-200-50-1-2 and G-200-50-1-3.

**Figure 9:**
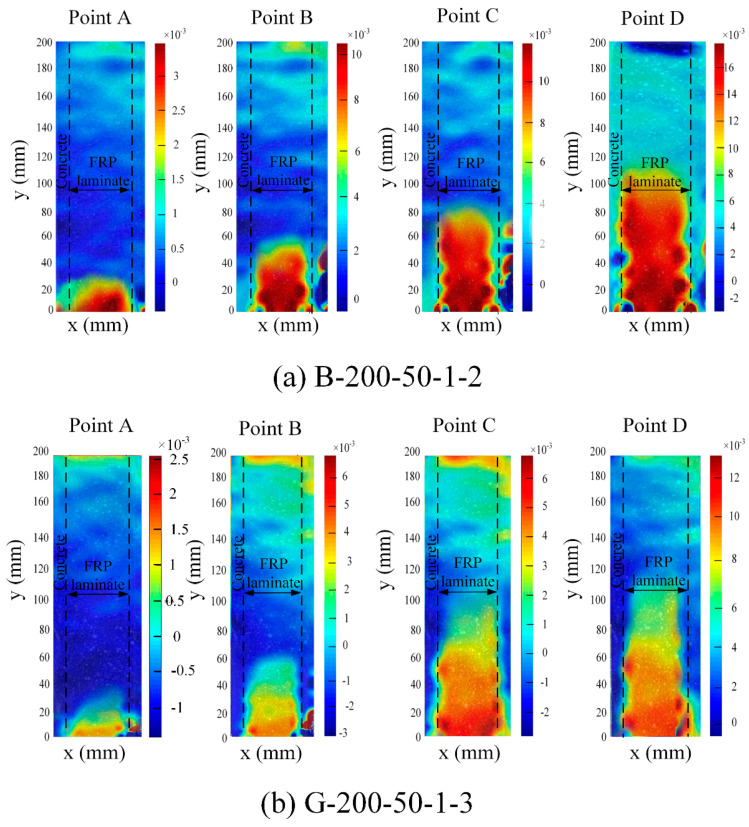
Strain contours of tested specimens under different load points.

The strain contours in the BFRP and GFRP laminates were obtained from Ncorr, an open-source 2D-DIC MATLAB software package, to analyze the characteristics of strain distributions during a double shear test. Figure 8 shows the points of load–slip curves for specimens G-200-50-1-3 and B-200-50-1-2 and Figure 9 shows the axial strain contours *ε_yy_* in FRP laminate at different loading. The dashed lines indicate the boundary of the FRP laminate. The strain in the BFRP laminate was nearly 35 mm as soon as the load moved to point A. With the increase in the load to point B, the strain further distributed 60 mm along the length of the FRP laminate. The magnitude of strain also increased quickly, and a large strain gradient emerged close to the loading end. The region of the peak strain gradient moved along with the length of the FRP laminate with the increase of load. At point C, the range of strain gradient was expanded to almost 80 mm. It can be seen at point D that the strain gradient slowly moved to the other end of the bonded joint, and the span increased to approximately to 120 mm, signifying a progressive debonding propagation between the FRP laminate and the concrete substrate as the slip at the loaded end increased.

**Figure 10:**
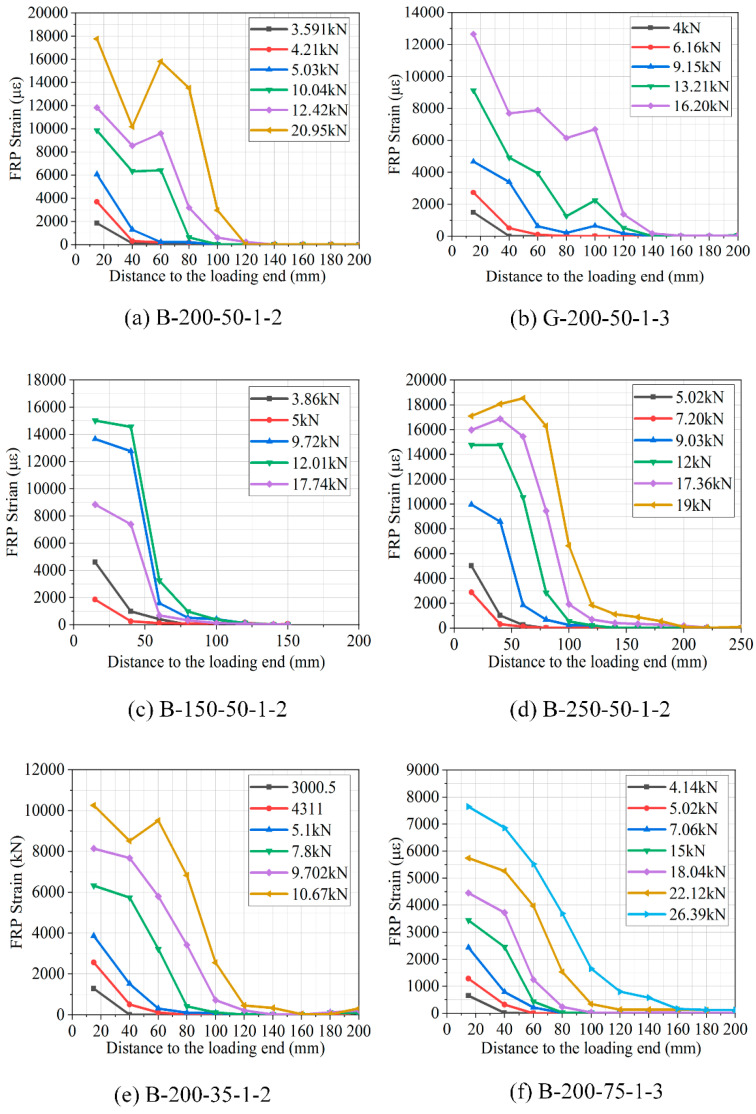

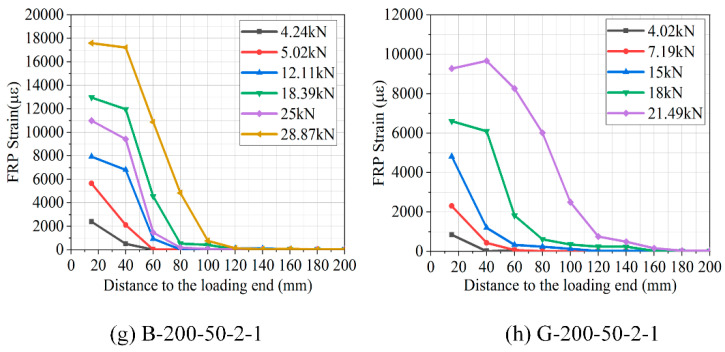
Strain distributions along FRP bonded length under different loading levels.

FRP strain distributions across the length of the bonded interface were used to determine the behavior of the FRP-to-concrete bonding interface [46,47]. Figure 10 displays the calculated FRP strain distribution of specimens under different loading levels obtained from the strain gauge. It can be seen from Figure 10 that the development of strain with different bonding lengths, different FRP widths, and different FRP thicknesses are almost identical for all tested specimens under different tensile loads. As the applied load was lower than the debonding load for specimens (e.g., B-200-50-1-1), the strain was mostly distributed around the bonded region of BFRP–concrete close to the loaded end. However, when the load was increased, the strains significantly developed throughout the loaded end of the FRP laminate when the ultimate was reached, it but gradually scaled linearly toward the free end, as shown in Figure 10a,b. Figure 10c,d displays the measured strain distributions along the FRP laminate for specimens B-150-50-1-1 and B-250-50-1-2 with different bonding lengths. The strains in the laminates show a linear curve trend when the load is still at a lower to average load level; however, they display a different pattern when the load has reached the ultimate load. As seen in the specimen with the longer bond length, the maximum BFRP strain has remained approximately constant, but the area in which this maximum BFRP strain has been attained continued to grow close to the free end, implying that debonding has propagated from the loading end toward the free end of BFRP laminate. Figure 10e,f depicts the measured strain distribution for specimens with different FRP widths. It is found that the maximum strain in the BFRP laminates increased greatly with the increase in load, forming very large BFRP strains over a much wider area after the ultimate load was achieved. Even though the recorded highest strains of the two specimens are similar, there seems to be a noticeable difference in the maximum load due to the different widths of BFRP. Figure 10g,h shows the comparison of the measured strain distributions along the bonded length of BFRP and GFRP laminates for specimens having two layers of FRP sheet strengthened with either GFRP or BFRP laminates under same loading respectively. Compared with the specimen reinforced with BFRP laminate, significant FRP strains developed only within a smaller region close to the loaded end before the ultimate load was reached but scaled linearly toward the free end.

### 3.5. Shear Stress Distribution along the BFRP and GFRP Bond Length

**Figure 11:**
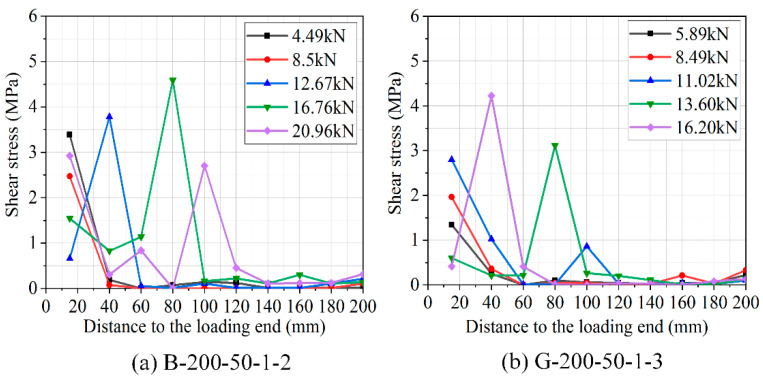

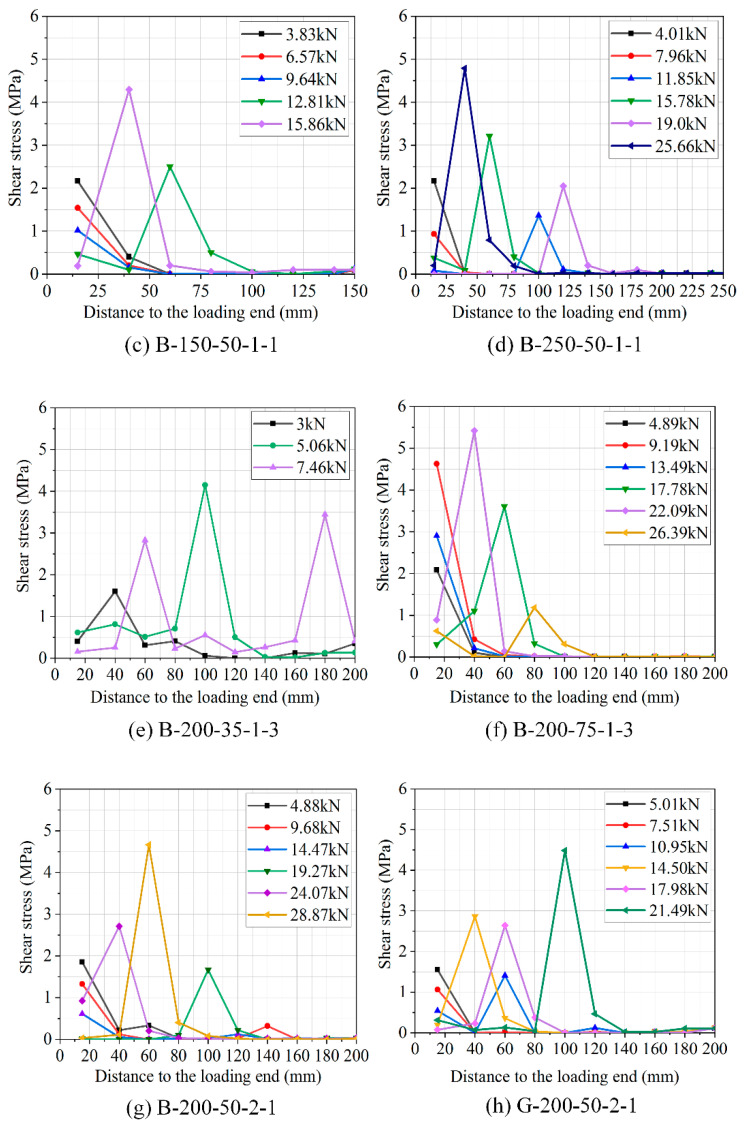
Shear stress distribution curves for specimens.

The shear stress distribution along the bonding length of BFRP and GFRP laminates was calculated in relation to the strain distribution shown in Figure 11. To describe the bond stress–slip behavior on the interfaces, several strain gauges have been fixed to the FRP laminates, which had a distance of 20 mm. The strain gauges allowed for recording the distribution of strains along the bonded interface. Based on the strain measurement taken from two adjacent strain gauges, the average bond shear stress of BFRP/GFRP laminate can be calculated as follows:

(1) $$\tau_{i}=\frac{\left(\varepsilon_{i}-\varepsilon_{i-1}\right)\times E_{f}t_{f}}{\Delta x}$$

where *τ_i_* is the average bond shear stress between BFRP or GFRP laminates and concrete at position *_i_*; *ε_i_* and *ε*_*i*−1_ are the strain values measured from the positions *i* and *i*−1 strain gauges; *t_f_* is the thickness of the BFRP/GFRP laminate; *E_f_* is the elastic modulus of the BFRP/GFRP laminate; and Δ*_x_* is the distance between strain gauges (*i*) and (*i*−1). The local slip can be expressed as:

(2) $$s_{i}=\frac{\Delta l}{2}\left(\varepsilon_{0}+2\overset{i=1}{\underset{j=1}{\sum }}\varepsilon_{j}+\varepsilon_{i}\right)$$

where *s_i_* is the local slip within the bond interface at position *i*; *ε*_0_ is the strain at the free end of BFRP/GFRP laminates; and *ε_j_* is the strain of the FRP laminate measured by the *j*th gauge.

The distribution of shear stress along the length of the FRP laminate for double shear specimens is presented in Figure 11. Upon loading, the bond stress near the loaded end of the BFRP/GFRP laminate tends to increase slowly as the load increases. The peak bond stress was found to shift away from the loaded end, along the bonded length of FRP laminate, reflecting debonding crack propagation. The peak bond stress for specimen B-200-50-1-2 in Figure 11a was at a distance 15 mm away from loading end once the applied load was only about 40% of its ultimate load. The shear stress was also very low over a distance of about 40 mm from the loading end. When the load applied was increased to 16.96 kN, the maximum bond stress moved to a distance of 80 mm toward the free end; the bond stress was lower again after a distance of about 100 mm to the free end. Figure 11 illustrates that (1) the shear stress in B-250-50-1-1 (Figure 11d) was zero once the load applied reaches the ultimate load. Although the shear stress at the loaded end in B-200-50-1-2 (Figure 11a) was still present, (2) the maximum bond stress of specimens moved along with the length of the FRP laminate from the loaded end when the applied load was increased. In the analysis of strain, this phenomenon supported the findings that the maximum effective shear stress region was expected to be longer than specimen B-200-50-1-2 but shorter than that of specimen B-250-50-1-1. The distribution of strain characteristics in the study agreed with the findings of Zheng XH [48].

### 3.6. Bond–Slip Relation

**Figure 12:**
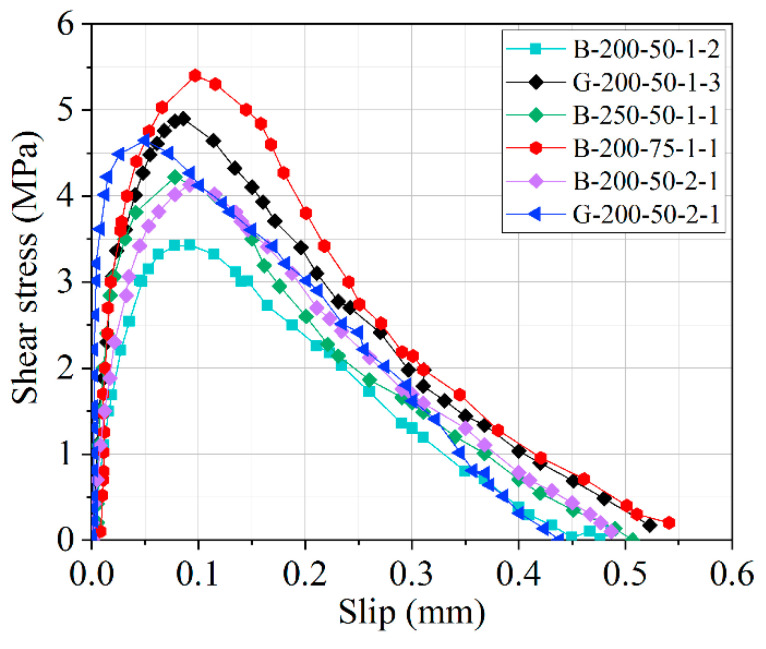
Bond–slip relationship of the tested specimens.

The bond–slip curves of the double shear specimens have been acquired through the use of Equations (1) and (2) plotted in Figure 12. From Figure 12, the shear stress and the corresponding slip initially increased linearly up to the peak value followed by minor nonlinear section before and after the peak stress. Once the peak stress has been reached, the curve descends slowly with an almost linear curve until the bond interface debonded completely or laminate rupture occurred. Although the initial stiffness of these specimens was nearly the same, the values of peak shear stress and the corresponding maximum shear slip were significantly different because of the various factors that influence them. These findings were consistent with the analysis of the texts described above.

**Figure 13:**
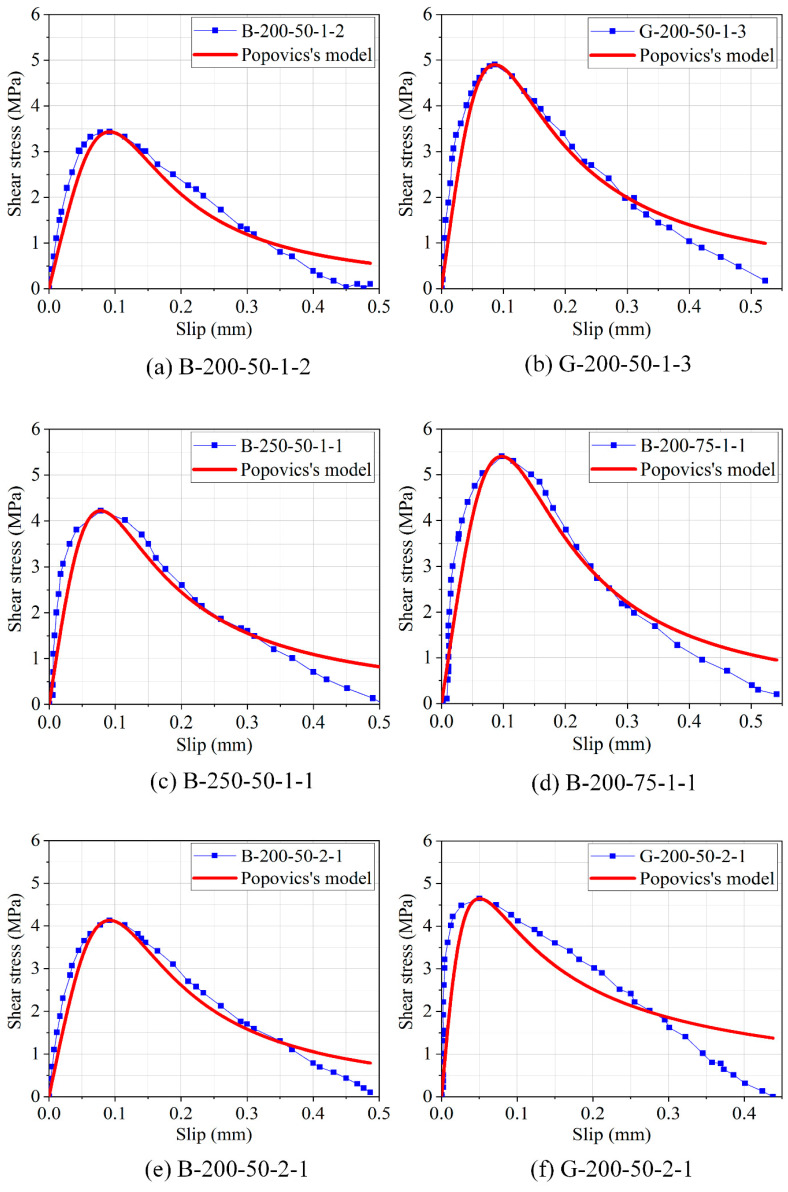
Bond–slip relationship of specimens fitted by Popovics’s model.

Numerous researchers [49,50] have implemented the equation proposed by Popovics [51] to predict the stress–slip relationship of the interface between the FRP laminate and concrete. In this present study, this equation was also suggested to determine the relationship between shear stress and slip that is given as:

(3) $$\tau \left(s\right)=\tau_{max}\left(\frac{S}{S_{0}}\frac{n}{\left(n-1\right)+{\left(S/S_{0}\right)}^{n}}\right)$$

in which *τ* means the shear stress, (MPa); *τ_max_* means the peak shear stress, (MPa); *s* means the local slip value (mm); *s*_0_ means the slip at the peak shear stress *τ_max_* (mm); and *n* is the constant that determines the shape (i.e., both ascending and descending branches) of the bond–slip curves based on experimental results. *τ_max_* and *s*_0_ values were derived directly from the experimental data. The results of regression coefficient (*n*) as well as the corresponding least squared (*R*^2^) have been listed in Table 2. Figure 13 displays the bond stress–slip curves of specimens under the influence of different FRP bond lengths, widths, and thicknesses. The maximum bond stress varied between 3.43 and 5.40 MPa, while the corresponding slip at peak shear stress was between 0.05 and 0.09 mm. The regression coefficient *n* increased from 1.83 to 2.66, indicating that these values have no clear relationship to FRP type. The inflection point seen for maximum shear stress (*τ_max_*) shows a tendency to increase when the bond width increases. The expected results of the Popovics’s equation are in agreement with the experimental data having the highest correlation coefficient.

### 3.7. Comparison of Experimental Results with Selected Bond–Slip Models

The behavior of the bond stress–slip curves of FRP-to-concrete bonding interface for double shear specimens (i.e., G-200-50-1-3 and B-200-50-1-2) were also compared to several other bond–slip models [49,52,53,54,55,56,57]. They can be classified into four categories with regard to their shapes [54]: i.e., models based on Popovic’s expression, elastic–plastic models, shear-softening models, and bilinear models. These models are mostly defined by the basic parameters such as the maximum shear stress and its corresponding slip as well as the ultimate slip.

**Figure 14:**
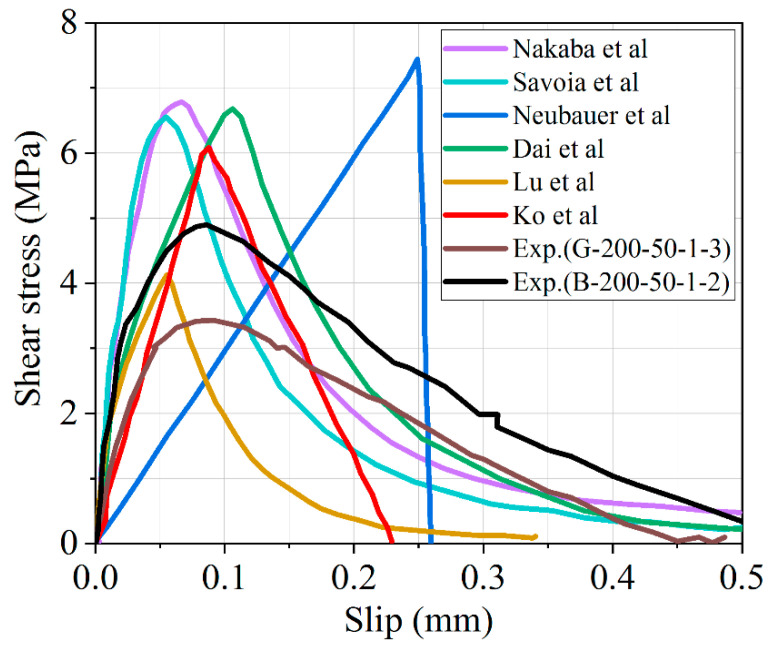
Comparison between the existing bond–slip models and experimental results (specimen G-200-50-1-3 and B-200-50-1-2).

As illustrated in Figure 14, there are six bond–slip models. The models mentioned above were chosen for calibration in order to determine the efficacy and accuracy of all the bond stress–slip models for double shear specimens. The maximum shear stress measured from the bond–slip models varied from 4.60 to 6.91 MPa, with the exception of Neubauer and Rostásy [56], which is very close to 7.41 MPa. The slip at the maximum shear stress is within the range of 0.06 to 0.24 mm. The models of Lu et al. [55] and Dai et al. [54] are all based on nonlinear fracture mechanics, yet they show distinct slips close to 0.1 mm. In addition, the maximum bond stress *τ_max_* of Dai’s model is approaching 7 MPa, which is much higher than the model of Lu, which is a little above 3 MPa. It is important to note that the model suggested by Nakaba et al. [49] has been based on Popovics’s model; meanwhile, Savoia et al.’s [57] model provides a very similar maximum shear stress of approximately 7 MPa and corresponding slips close to 0.06 mm. The peak bond stress of the specimens B-200-50-1-2 and G-200-50-1-3 are 3.43 and 4.89 MPa less than the maximum shear stress presented by the literature models. Based on the experimental results, the bond–slip models overestimate the maximum shear stress not only for B-200-50-1-2 but also for G-200-50-1-3. The overestimation of experimental results could be attributed to the discrete nature of concrete cracks and the heterogeneity of concrete. However, the slip at maximum shear stress *τ_max_* is nearly identical to the average value of the other bond stress–slip models other than Neubauer and Rostásy.

## 4. Conclusions

An experimental study on the mechanical properties and the factors that influence the performance of the bond between basalt fiber (BFRP) and glass fiber (GFRP) laminates strengthened concrete is discussed. A 2D-DIC (digital image correlation) procedure was also utilized for calculating strains on the surface of FRP–concrete test specimens in addition to the strains recorded by the conventional strain-measuring device (strain gauge). Thirty-six twin specimens were prepared and were subjected to the double shear loading, with testing parameters such as bond length, width, and thickness. Variations in failure mode, load–slip relationship, strain distribution in FRP laminate, shear stress distribution, and shear stress and slip were measured and analyzed in detail. The results from the experiments can be used to draw the following conclusions:

1. The failure mode of BFRP/GFRP–concrete samples from the double shear testing has been attributed mostly to the state of the bonding surface of the concrete block. If the bonding surface of concrete consisted of aggregate and mortar, several debonding modes, such as debonding in concrete, debonding at an adhesive–concrete interface, and debonding at the adhesive–FRP laminate interface can happen. Even though the ultimate load is affected by the characteristics of FRP, concrete, and adhesive, their impact on the bond behavior comparative to one another remains variable and uncertain. In addition, the main debonding mode of samples failure has been associated with the distribution density of aggregate within the bonding surface of FRP and concrete.
2. FRP width has a significant impact on the bonding behavior of the BFRP laminate–concrete interface. An increase in FRP width results in improving the maximum load-carrying capacity with higher stiffness and greater ductility. This may be because shear stresses are distributed over a larger bonded area.
3. The thickness of FRP laminate has the same influence on the interfacial behavior of BFRP and GFRP samples significantly. Increasing the thickness to double layers of FRP sheet greatly increase the bonding interface of the load-bearing capacity. With the increase in FRP thickness and slip, the ultimate load increased significantly until failure. However, GFRP specimens recorded lower debonding load compared with BFRP specimens.
4. The bond length has a distinct influence on the bond behavior. Increasing the length of BFRP laminate above 200 mm results in higher load capacity of the interface between FRP laminate–concrete. The slip can get enhanced with the increasing bond length when the length is less than the maximum effective bonding length.
5. The values of peak shear stress and the corresponding maximum shear slip were significantly different because of the various factors’ influences on them.

## Figures and Tables

**Table 1:** Parameters of investigation and results from the bond test.

Specimen Name	Type of FRP	No. of Sheets	Bond Length (mm)	Width (mm)	Ultimate Load (kN)	Avg Ultimate Load (kN)	Failure Mode
B-200-50-1-1	BFRP	1 layer	200	50	17.00	18.03	DC
B-200-50-1-2	BFRP	1 layer	200	50	20.96		DC
B-200-50-1-3	BFRP	1 layer	200	50	16.05		DC
B-150-50-1-1	BFRP	1 layer	150	50	15.86	18.25	DC
B-150-50-1-2	BFRP	1 layer	150	50	17.95		AD
B-150-50-1-3	BFRP	1 layer	150	50	21.15		DC
B-250-50-1-1	BFRP	1 layer	250	50	25.66	22.12	DC
B-250-50-1-2	BFRP	1 layer	250	50	22.00		DC
B-250-50-1-3	BFRP	1 layer	250	50	18.68		AC
B-200-35-1-1	BFRP	1 layer	200	35	10.55	9.56	AD
B-200-35-1-2	BFRP	1 layer	200	35	10.67		AD
B-200-35-1-3	BFRP	1 layer	200	35	7.47		DC
B-200-75-1-1	BFRP	1 layer	200	75	29.86	27.94	DC
B-200-75-1-2	BFRP	1 layer	200	75	27.57		DC
B-200-75-1-3	BFRP	1 layer	200	75	26.39		DC
B-200-50-2-1	BFRP	2 layers	200	50	28.87	24.05	DC
B-200-50-2-2	BFRP	2 layers	200	50	21.49		DC
B-200-50-2-3	BFRP	2 layers	200	50	27.90		DC
G-200-50-1-1	GFRP	1 layer	200	50	15.74	15.95	FR+DC
G-200-50-1-2	GFRP	1 layer	200	50	15.93		FR
G-200-50-1-3	GFRP	1 layer	200	50	16.20		FR
G-200-50-2-1	GFRP	2 layers	200	50	21.49	23.17	DC
G-200-50-2-2	GFRP	2 layers	200	50	25.65		DC
G-200-50-2-3	GFRP	2 layers	200	50	25.37		DC

Note: DC represents debonding in concrete; AD means adhesive–FRP debonding; AC is adhesive–concrete debonding and FR means FRP rupture.

**Table 2:** Experimental and fitted results of bond stress–slip relationship.

Specimen Number	*τ_max_* (MPa)	*S*_0_ (mm)	Regression Coefficient *n*	Correlation Coefficient *R*^2^
B-200-50-1-2	3.43	0.081	2.66	0.932
B-250-50-1-1	4.21	0.077	2.32	0.878
B-200-75-1-3	5.40	0.097	2.54	0.901
B-200-50-2-1	4.13	0.091	2.53	0.947
G-200-50-1-3	4.89	0.085	2.34	0.929
G-200-50-2-1	4.64	0.050	1.83	0.648
